# Deciphering the flapping frequency allometry: unveiling the role of sustained body attitude in the aerodynamic scaling of normal hovering animals

**DOI:** 10.1242/bio.061932

**Published:** 2025-03-14

**Authors:** Jeremy Pohly, Chang-kwon Kang, Hikaru Aono

**Affiliations:** ^1^ Department of Mechanical and Aerospace Engineering, University of Alabama in Huntsville, Huntsville, Alabama 35899, USA; ^2^ Department of Mechanical Engineering and Robotics, Shinshu University, Ueda, Nagano 3868567, Japan

**Keywords:** Normal hovering, Animal flight, Scaling

## Abstract

Hovering flight helps facilitate feeding, pollination, and courtship. Observed only in smaller flying animals, hover kinematic characteristics are diverse except for the decreasing flapping frequency with the animal size. Although studies have shown that these wing patterns enable distinct unsteady aerodynamic mechanisms, the role of flapping frequency scaling remains a source of disagreement. Here we show that negative allometry of the flapping frequency is required to sustain body attitude during hovering, consistent with experimental data of hovering animals, from fruit flies to hummingbirds, reported in the literature. The derived scaling model reveals that the lift coefficient and reduced frequency remain invariant with mass, enabling leading-edge vortex formation and wake-capture for a wide range of fliers to hover.

## Teaser

**Summary:** The frequency allometry from the sustained body attitude provides a missing constraint to the theoretical scaling model of the aerodynamics of normal hovering animals.

## INTRODUCTION

Hovering stands as a pivotal flight mode in the animal kingdom, enabling essential functions like feeding, pollination, and mating (Anderson et al., 2005). In particular, normal hovering aminals employ a distinctive wing-flapping pattern in a nearly horizontal stroke plane with symmetrical half-strokes to maintain their equilibrium in still air (Weis-Fogh, 1973). Other hovering modes are the asymmetric hover and wind-hover modes. Bats and birds that are capable of hovering – other than hummingbirds – show this mode where the stroke plane is more titled and the wings are flexed during the upstroke, producing asymmetric lift generation (Håkansson et al., 2015; Norberg et al., 1993). Finally, in wind hovering the animal leverages the surrounding wind (Penn et al., 2022).

While normal hovering is observed across a wide range of animal sizes, from tiny fruit flies to small birds such as hummingbirds, hovering is also one of the most energy-demanding forms of locomotion (Weis-Fogh, 1972). Without relying on the forward motion (Berman and Wang, 2007) and the steady wing aerodynamic mechanisms as with an airplane, hovering animals benefit from unsteady aerodynamic mechanisms (Shyy et al., 2013); the formation of vortices from the thin leading-edge of their wings (Bomphrey et al., 2017; Dickinson et al., 1999; Ellington et al., 1996), wake-capture (Dickinson et al., 1999) through subsequent wing-wake interaction, and clap-and-fling (Weis-Fogh, 1973).

Despite extensive investigations spanning nearly a century by scientists and engineers (Hill, 1950), understanding how hovering flight scales with animal size remains incomplete. Notably, smaller insects exhibit faster flapping motion than larger birds during hovering. While this negative allometry of flapping frequency is well recognized, a consensus on the form and explanation of this scaling relationship remains elusive (Azuma, 2006; Deakin, 2010; Dudley, 2000; Greenwalt, 1962; Weis-Fogh, 1977).

Lacking a physics-based scaling relation for flapping frequency, previous models for hover scaling (Azuma, 2006; Norberg, 2006; Pennycuick, 1975; Jensen et al., 2024) depended on a questionable simplification: assuming the lift coefficient in hovering to be both constant and the maximum achievable value. However, lift coefficient is not at maximum during hovering as higher values have been reported in other flight modes (Dudley and Ellington, 1990a; Willmott and Ellington, 1997). Also, the constant lift coefficient simplification remains unjustified and untested against empirical data.

Here, we consider and derive a physics-based scaling relation for the flapping frequency of normal hovering animals. This derivation is driven by the hypothesis that negative allometry of the flapping frequency is required to sustain body attitude during hovering. These relations are tested against a total of *N*_ob_=171 experimental observations of *N*_sp_=27 insect and hummingbird species in normal hover flight (Chai and Millard, 1997; Cheng and Sun, 2016; Dudley and Ellington, 1990a; Ellington, 1984a,b; Fry et al., 2005; Groom et al., 2018; Kolomenskiy et al., 2019; Lehmann and Dickinson, 1997; Liu et al., 2024; Meng and Sun, 2015; Willmott and Ellington, 1997), spanning a range of masses O(10^−1^)−O(10^4^) mg (Data S1). The existing theoretical scaling model for the flapping wing aerodynamics of normal hovering animals is underdetermined as the number of morphological, kinematic, and energetic constraints and relations is insufficient to fully define the physics governing hovering. This paper aims to refine the model by introducing a dynamic constraint, leading to a scaling relation for the flapping frequency. This additional constraint renders the hover scaling model determinate.

## RESULTS AND DISCUSSION

### Isometry of the wing shape and body

Before deriving the frequency allometry, we tested if the wing length and surface area were isometric. The surface-area-to-volume ratio scaling suggests that wing length (*R*) and area (*S*) increase isometrically with mass according to *R*∼*m*^1/3^ and *S*∼*m*^2/3^. The ordinary least-squares (OLS) scaling and the phylogenetic generalized least-sqaures (PGLS) scaling for the wing length based on the experimental data for the considered hovering animals (Fig. 2A) indeed shows an isometric behaviour of 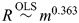 and 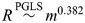 (Fig. 2B and Tables 1 and 2). The complete dataset can be found in Dataset S1. The wing area allometry of 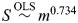 and 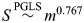 (Fig. 2C and Tables 1 and 2) is slightly higher than the theoretical 2/3 exponent. This is due to the known hyperallometry of hummingbird wing area (Skandalis et al., 2017) with an exponent estimated between 1.1 and 1.3 (Greenwalt, 1962). This departure from isometry is surmised as necessary to maximize aerial performance and minimize flight costs as hummingbird mass increases (Skandalis et al., 2017). Excluding the hummingbird data results in an isometric scaling of 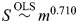 and 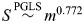 (Fig. S3 and Tables S4, S5).

**Fig. 1.**
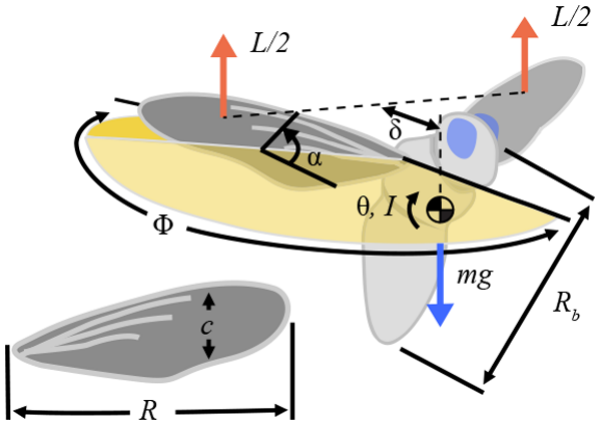
**A schematic illustrating the key hover parameters considered in this study.** Wing length *R*, mean chord length *c*, horizontal distance between centers of mass and pressure δ, body length *R*_b_, flap amplitude Φ, wing angle of attack α, body pitch angle θ, body inertia *I*, total mass *m*, aerodynamic lift *L*, and gravitational acceleration *g*.

**Fig. 2.**
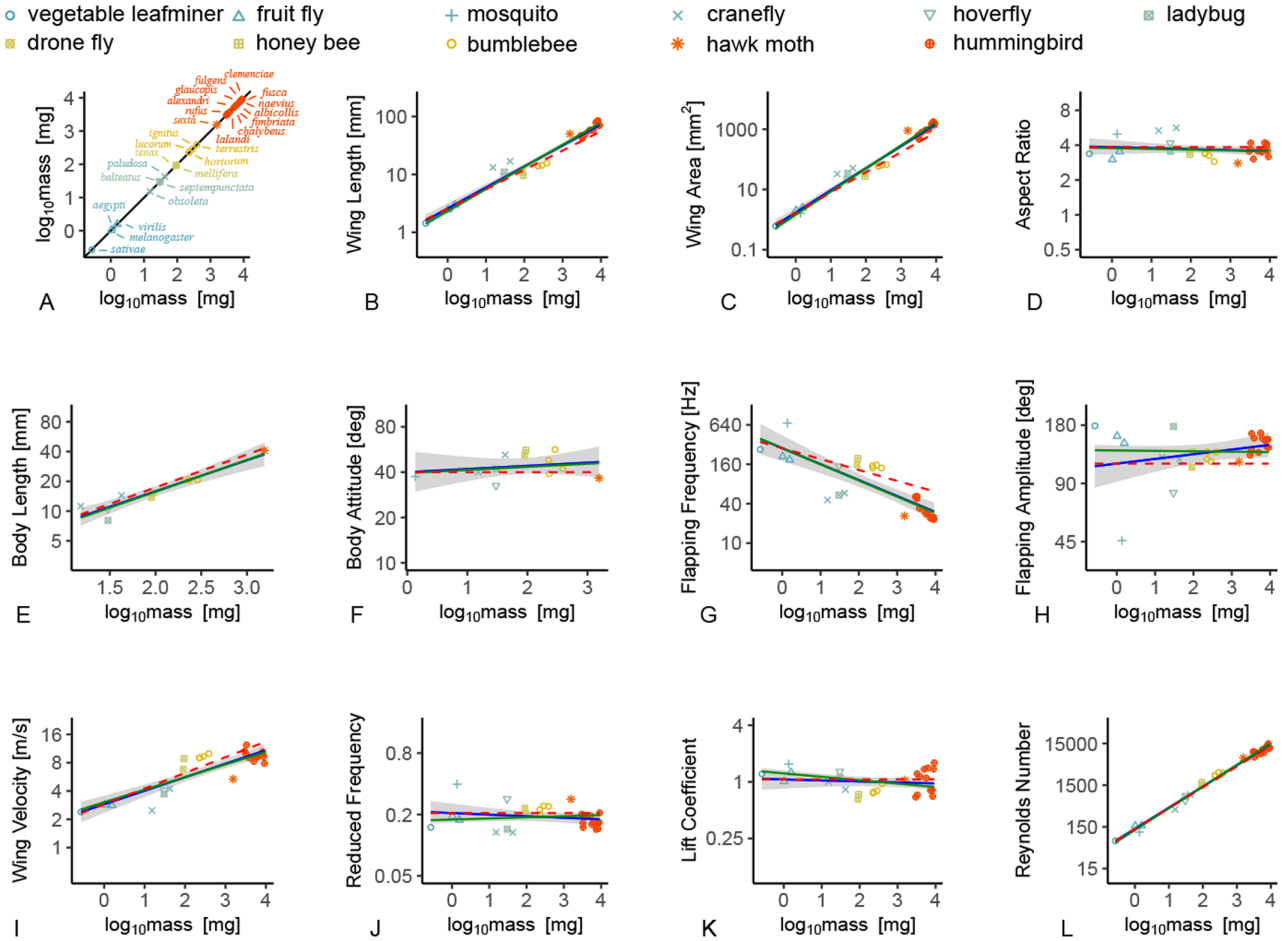
**Dimensional and non-dimensional allometric relationships in normal hovering animals.** (A) Considered insect and bird species and the OLS and PGLS regressions for (B) the wing length *R*, (C) wing area *S*, (D) aspect ratio *AR*, (E) body length *R*_b_, (F) body attitude (mean body pitch angle θ_m_), (G) flapping frequency *f*, (H) flapping amplitude Φ, (I) wing velocity *U*, (J) reduced frequency *k*, (K) lift coefficient *C*_L_, and (L) Reynolds number *Re* with mass *m* in hovering flight. Shaded regions, 95% confidence intervals of the OLS regression. Blue and green solid lines, OLS and PGLS regression. Red dashed lines, theoretical scaling. Tables 1 and 2 show the numeric results.

**Table 1.** Theoretical and empirical OLS scaling results for normal hovering animal parameters

	*N*_ob_	*N*_sp_	γ_theory_	γ_OLS_	log_10_β_OLS_	*r* ^2^_OLS_	*P*_OLS_	95% CI
*R*	171	27	1/3	0.363	0.412	0.957	<0.001	[0.332, 0.394]
*S*	171	27	2/3	0.734	0.238	0.969	<0.001	[0.681, 0.787]
*AR*	171	27	0	−0.009	0.585	−0.013	0.424	[−0.030, 0.013]
*R*_b_	42	10	1/3	0.314	0.572	0.876	<0.001	[0.224, 0.404]
θ_m_	30	12	0	0.021	1.602	−0.050	0.508	[−0.047, 0.090]
*f*	143	27	−1/6	−0.244	2.450	0.668	<0.001	[−0.313, −0.175]
Φ	143	27	0	0.024	2.057	0.048	0.141	[−0.009, 0.057]
*U*	143	27	1/6	0.144	0.461	0.762	<0.001	[0.111, 0.176]
*k*	143	27	0	−0.015	−0.685	−0.003	0.345	[−0.048, 0.017]
*C*_L_	143	27	0	−0.011	0.026	−0.023	0.531	[−0.045, 0.024]
*Re*	143	27	1/2	0.510	2.124	0.989	<0.001	[0.489, 0.532]

CI denotes confidence intervals. *R*, wing length; *S*, wing area; *AR*, aspect ratio; *R*_b_, body length; θ_m_, body attitude (mean body pitch angle); *f*, flapping frequency; Φ, flapping amplitude; *U*, wing velocity; *k*, reduced frequency; *C*_L_, lift coefficient; and *Re*, Reynolds number.

**Table 2.** Theoretical and empirical PGLS scaling results for normal hovering animal parameters

	*N*_ob_	*N*_sp_	γ_theory_	γ_PGLS_	log_10_β_PGLS_	*P*_GPLS_
*R*	171	27	1/3	0.382	0.367	<0.001
*S*	171	27	2/3	0.767	0.164	<0.001
*AR*	171	27	0	−0.007	0.576	0.695
*R*_b_	42	10	1/3	0.321	0.553	<0.001
θ_m_	30	12	0	0.021	1.596	0.589
*f*	143	27	−1/6	−0.248	2.452	<0.001
Φ	143	27	0	−0.002	2.124	0.941
*U*	143	27	1/6	0.134	0.482	<0.001
*k*	143	27	0	0.011	−0.748	0.699
*C*_L_	143	27	0	−0.036	0.090	0.238
*Re*	143	27	1/2	0.521	2.102	<0.001

*R*, wing length; *S*, wing area; *AR*, aspect ratio; *R*_b_, body length; θ_m_, body attitude (mean body pitch angle); *f*, flapping frequency; Φ, flapping amplitude; *U*, wing velocity; *k*, reduced frequency; *C*_L_, lift coefficient; *Re*, Reynolds number.

The isometry of the aspect ratio based on the wing length and area results in *AR*∼*R*^2^*/S*∼*m*^2/3^*/m*^2/3^∼*m*^0^, which is confirmed by the OLS scaling of 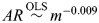 and PGLS scaling of 
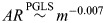
 (Fig. 2D and Tables 1 and 2). The body length *R*_b_, moment of inertia *I* and the distance δ between the centers of pressure and gravity are less documented in the literature. Assuming isometry for the distance δ between the centers of pressure and gravity and the body length *R_b_*, we have δ∼*R_b_*∼*m*^1/3^, consistent with the experimental observation of body length scaling as 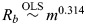 and 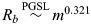 (Fig. 2E and Tables 1 and 2). Based on these assumptions, the theoretical scaling for the body moment of inertia is derived as *I*∼*mR_b_*^2^∼*m*^1^*m*^2/3^∼*m*^5/3^. However, this theoretical scaling cannot be validated against experimental data due to the lack of measured values for body inertia in the literature.

### Flapping frequency allometry for hover equilibrium

A longstanding question in the study of animal flight is how flapping frequency relates to body size, given the clear observation that smaller hovering animals flap more quickly than larger ones. Despite this consistent pattern, a unified explanation for the scaling of flapping frequency remains elusive. Flight muscles activate the wing motion (Gau et al., 2023; Molloy et al., 1987) and, as such, earlier studies (Greenwalt, 1962; Hill, 1950; Rashevsky, 1960; Weis-Fogh, 1977) proposed that *f*∼*m*^−1/3^ by modeling the muscles as mechanical oscillators. However, observations of birds and insects did not perfectly coincide (Weis-Fogh, 1977); data regression and dimensional analysis suggested relations of *f*∼*m*^−0.383^ by Greenwalt (1962), and *f*∼*m*^−0.24^ by Dudley (2000). Finally, Deakin (2010) argued that *f*∼*m*^−1/6^ is the correct allometry from the dimensional perspective and empirically matched the data by Byrne et al. (1988).

A potential cause for the variance in the flapping frequency allometry is the blend of the hovering and non-hovering motion measurements in these datasets (Dudley, 2000; Greenwalt, 1962). The unsteady aerodynamic mechanisms enabling hover are qualitatively different from non-hover flight dynamics (Shyy et al., 2013). As such, a universal frequency allometry may not exist due to the diversity among a wide range of flying animal morphology, kinematics, and flight modes. In addition, earlier experimental observations, which mostly relied on acoustic methods, faced the challenge of accurately measuring the flapping frequency. While these acoustic methods were state-of-the-art at the time, modern advancement in high-speed photography provides a more accurate and reliable approach to determine flapping frequency during hovering.

That said, the relation *f*∼*m*^−1/6^ can also be derived for hover flight, if a constant lift coefficient can be assumed (Azuma, 2006; Norberg, 2006; Pennycuick, 1975). However, the constant lift coefficient simplification has not been justified nor tested against data.

Here, we consider attitude dynamics to derive the frequency scaling without relying on the constant lift coefficient assumption. Eqn 2 is nonlinear through *C*_L_, given by Eqn 3 and an exact solution is unknown. Instead, we use scaling arguments for the body pitch amplitude θ_a_. If the body pitch is harmonic (Eberle et al., 2015) with a phase lag ϕ and mean pitch of θ_m_, i.e. θ=θ_a_cos(2π*ft*+ϕ)+θ_m_, then d^2^θ/d*t*^2^=−4π^2^θ_a_ *f*^2^cos(2π*ft*+ϕ). The time scales as *t*∼1/*f* as the main time scale associated with the aerodynamic pitching moment is 1/*f*. In general, the pitch acceleration scales with the square of the frequency, such that d^2^θ/d*t*^2^∼−θ_a_ *f*^2^.

The lift scales with the weight during hover equilibrium, i.e. *L*∼*mg*. Furthermore, with *I*∼*m*^5/3^, and δ∼*m*^1/3^, i.e. I*∼*m*^0^, and δ*∼*m*^0^, the body pitch amplitude scaling reduces to θ_a_∼g/(*R*_b_*f*^2^), implying that the body pitch amplitude scales with the ratio of the gravitational acceleration to the angular acceleration based on the body length.

Due to geometric and mechanical restrictions, the body pitch angle variation is constrained and typically limited to less than 180° Then, θ_a_∼*m*^1/3^*f*^−2^∼*m*^0^. This is indirectly supported by the mean pitch amplitude scaling of 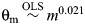 and 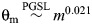 (Fig. 2F and Tables 1 and 2) as θ_a_ is rarely reported. The flapping frequency scaling follows as *f*∼*m*^−1/6^, close to the empirical OLS scaling of 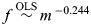 and the PGLS scaling of 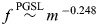 (Fig. 2G and Tables 1 and 2). Note again that the empirical OLS and PGLS scaling are based only on hovering flight and not on mixed flight modes.

The same frequency scaling was derived from data fitting and dimensional analysis by Deakin (2010) or by assuming a constant lift coefficient (Azuma, 2006; Norberg, 2006; Pennycuick, 1975). The presented derivation highlights a physics-based argument for the negative allometry of the flapping frequency required to maintain both the weight and the attitude during hovering. In particular, if the exponent of the frequency scaling was less than −1/6, then smaller insects would experience extremely rapid body pitch rotations and angles, making them unstable. Conversely, if the exponent was greater than −1/6, then larger insects and birds would experience this instability. By flapping at *f*∼*m*^−1/6^ the corresponding body scale provides appropriate moment of inertia to keep the body attitude mass invariant.

### Dynamically similar hovering mechanism

The flapping and pitching amplitudes of the wing are also bound by geometric and mechanical restrictions. As such, the flapping angle is independent of mass 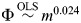 and 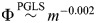 (Fig. 2H and Tables 1 and 2). The pitching amplitude α is far less frequently reported in the literature. Nevertheless, flapping wing aerodynamics theories and experimental observations suggest that α is around 45° for optimal lift generation during the translational phase of each stroke during hovering (Dickinson et al., 1999; Shyy et al., 2013), also independent of mass.

The wing tip velocity scaling for hovering becomes *U*=2Φ*fR*∼2*m*^0^*m*^−1/6^*m*^1/3^∼*m*^1/6^. A good agreement is shown here between the theoretical and empirical scaling for the mean tip velocity in hovering 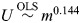 and 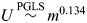 (Fig. 2I and Tables 1 and 2). Despite the inverse relationship between frequency and mass, wing velocity exhibits a positive allometry due to the faster increase in *R*. The positive allometry of wing velocity helps produce sufficient lift for heavier animals.

The reduced frequency *k* is inversely proportional to the flapping amplitude Φ and the aspect ratio *AR*. Although these two non-dimensional parameters show some subtle variations, their magnitude remains in the same order of magnitude with respect to a change in the animal size. A consequence is that the reduced frequency *k*∼π/(2Φ*AR*)∼π/(2*m*^0^*m*^0^)∼*m*^0^ does not scale with mass either: 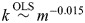 and 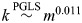 (Fig. 2J and Tables 1 and 2). This implies that the unsteadiness of the flow around hovering animals is about the same and size invariant. Near constancy of *k* is due to the balance between the dynamic stall timescale ∼*c*/*U* and the flapping period ∼1/*f*. This balance ensures a similar flow unsteadiness across hovering species. In addition, *k*=π/(2Φ*AR*) for hovering flight is the inverse of the stroke-arc to chord ratio (Shyy et al., 2013), which is also the ratio between the transient timescale associated with the dynamic stall and the flapping period (Wang, 2005).

The dynamic stall through the leading-edge vortices (LEVs) and the subsequent wake capture are key unsteady flow features, used to enhance lift generation for flapping wings (Ellington et al., 1996; Shyy et al., 2013). These LEVs are responsible for nearly half of the lift for hovering insects (Dickinson et al., 1999; Van Den Berg and Ellington, 1997) and a quarter of the lift in hummingbirds (Warrick et al., 2009). To benefit from the dynamic stall, these two timescales need to be in balance such that 0.15<*k*<0.25 (Wang, 2005), which is shown by the experimental data (Fig. 2J).

The hover lift coefficient is defined from Eqn 1 as *C*_L_=2 m*g*/(ρ*U*^2^*S*). This definition includes all unsteady aerodynamic effects as *mg*=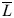 in hover. *C*_L_ is also invariant of mass, which can be seen by inserting *f*∼*m*^−1/6^, Φ∼*m*^0^, *R*∼*m*^1/3^, and *S*∼*m*^2/3^ in Eqn 1, i.e. *C*_L_∼*m*/(*U*^2^*S*)∼*m*/(*m*^1/3^*m*^2/3^)=*m*^0^. Theoretically, the lift coefficient *C*_L_ is invariant with mass, which is broadly consistent with experimental observations (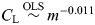 and 
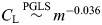
) (Fig. 2K and Tables 1 and 2). The invariance of the lift coefficient, the reduced frequency, and the remaining non-dimensional parameters other than the Reynolds number with mass suggest that the unsteady lift production mechanism is dynamically similar. This implies that normal hovering animals benefit from the same unsteady aerodynamic mechanism despite the variations in their morphology and wing motion. The invariance of the lift coefficient and the dynamic similarity are not trivial results, particularly because of the strong variation of the Reynolds number with mass as discussed in the section ‘Reynolds number’. The clap-and-fling follows the same scaling (Weis-Fogh, 1977). These suggest that the lift production mechanisms in the hovering flight are dynamically similar, despite the differences in the details of the wing motion and the morphology.

### Reynolds number

On the other hand, the Reynolds number exhibits a pronounced dependence on mass. The isometry of the wing and the −1/6 exponent of the frequency results in *Re*∼*ρUc*∼*ρm*^1/6^*m*^1/3^ ∼*m*^1/2^. A good agreement is evident between the theoretical and empirical scaling 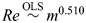 and 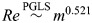 (Fig. 2L and Tables 1 and 2). These results signify a nearly perfect allometric relation with the 1/2 power scaling. This close agreement further supports the validity of the −1/6 exponent of the flapping frequency allometry. Any alternative allometric coefficient for the flapping frequency would inevitably lead to a Reynolds number scaling exponent that deviates from the observed square-root behaviour.

Within the scope of this study, we have shown theoretically that all dimensionless parameters in Eqn 3 are invariant with mass, except for the Reynolds number (*Re*). This theoretical invariance is supported by the statistical results for *AR*, *k*, and Φ, as shown in Tables 1 and 2. Both OLS and PGLS regression analysis for these parameters indicate weak trends (exponents close to zero) that are not statistically significant (*P*>0.001). Furthermore, we cannot experimentally show that α, θ_a_, I*, and δ* are invariant with mass as data for these parameters are limited. That said, the theoretical invariance of these four terms is implicitly supported by the Reynolds number scaling, which shows close agreement between the theoretical prediction and the statistical result.

The invariance of the remaining dimensionless parameters suggests that the hovering mechanism may be Reynolds number-independent in the considered range. Based on this argument, we can formulate a hypothesis that the main unsteady lift enhancement mechanism, i.e. the formation of the leading-edge vortices, is weakly dependent on the Reynolds number. A potential mechanism that supports this hypothesis is that the thin and sharp leading edge shapes of the animal wings lead to the leading edge vortex formation. Because of the small radius of curvature of the leading-edge, the pressure gradient of the flow is large. As a consequence, the flow separates from the leading edge, forming a coherent vortex structure in the low Reynolds number regime. This is a different mechanism than for a modern aircraft, where the airfoil shape is streamlined. Flow separation on such a streamlined shape is typically due to the detachment of the boundary layer from the surface, typically a high Reynolds number flow feature. Furthermore, the Reynolds number becomes the key parameter that describe the boundary layer characteristics. A similar observation was reported by Lentink and Dickinson (Lentink and Dickinson, 2009) who stated that “the mechanism responsible for LEV stability is not dependent on Reynolds number” within the range of 100<*Re*<14,000.

Another interesting observation is that the Reynolds number of flapping and fixed wings in forward flight also has a 1/2 allometry coefficient (Liu, 2006). While the exact underlying aerodynamic mechanisms behind these two observations are currently unknown, the Reynolds number scaling illustrates an exciting trend evinced by flying animals and vehicles.

### Conclusion

The current theoretical scaling model for the flapping wing aerodynamics of normal hovering animals in normal hover is underdetermined because the number of morphological, kinematic, and energetic constraints and relations is less than the number of physical variables and parameters describing hovering flight. The primary aim of this paper was to augment the scaling model with a dynamic constraint, which results in a scaling relation for the flapping frequency. This additional constraint makes the hover scaling model determinate.

The derived scaling law for flapping frequency with the allometric coefficient −1/6 aligns reasonably well with the OLS regression with the exponent of −0.244 and the PGLS regression with the exponent of −0.248. While there is some deviation between the theoretical and observed exponents, we observe that across a more diverse dataset – spanning both unspecified flight modes for various insects and bats in near-hover conditions – the observed scaling relationship remains consistent. In fact, the allometric coefficient for this extended dataset is closer to the theoretical value as the OLS regression is 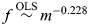 and the PGLS regression is 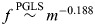.

That said, we do not expect to see an exact match between the theoretical scaling model and the experimental observations as a scaling model cannot capture all the salient details of the animal morphology and unsteady aerodynamics.

We made a simplifying assumption that the detailed features, such as halteres and elytra, have a secondary effect on unsteady aerodynamics compared to the primary mechanisms under consideration. These features contribute significantly less due to their smaller surface area and limited flapping amplitude relative to the wings. Since lift magnitude is proportional to wing area and the square of the reference velocity, the lift generated by small halteres and elytra with low flapping amplitudes (and thus reduced reference velocity) is negligible compared to the wings (Oh et al., 2020). Moreover, this study focuses on deriving general scaling relationships applicable to all normal hovering animals. Features like halteres or elytra, which are specific to certain species, are unlikely to be essential for enabling normal hovering across all species. While variations in morphology, wing shape, and specialized adaptations do exist, these species-specific details are beyond the scope of our physics-based scaling framework, which aims to identify universal trends rather than address individual intricacies.

The additional slight deviations from the theoretical scaling results could also stem from several factors, such as wing flexibility, hyperallometric effects (e.g. hummingbirds), and measurement uncertainty. Since our model assumes rigid wings, wing flexibility effects will introduce additional time scales due to structural frequencies and impact aerodynamic performance in non-trivial ways [see Kang et al. (2011)]. In addition, the regression analysis of these data without hyperallometry (hummingbirds) shows a closer agreement between the theoretical scaling and the experimental data (Fig. S3 and Tables S4, S5). In particular, the frequency allometric coefficients without hummingbirds are −0.171 (OLS) and −0.233 (PGLS), which are closer to the theoretical −1/6 than −0.244 (OLS) and −0.248 (PGLS) with hummingbirds. The *P*-value is slightly higher (0.038 for the OLS and 0.016 for the PGLS), likely due to the smaller sampler size. Further studies exploring wing flexibility and hyperallometric effects remain important area for future research.

Despite these assumptions, the proposed scaling relations offer valuable insights into the fundamental principles governing the normal hovering flight across a wide range of species. In addition, the linear and the phylogenetic regression analyses of the normal hovering motions of flying animals provide a comprehensive validation of the scaling law within the constraints of the available experimental data.

## MATERIALS AND METHODS

### Non-dimensional parameters for hovering

In an equilibrium hover flight lift balances the weight. To derive the scaling relations, we consider an animal with mass *m* as:
(1)

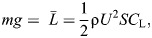
where *g* is the gravitational acceleration, *L* is the lift, 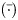 is the cycle-averaging operator, ρ is the air density, *U*=2Φ*fR* is the mean wing tip velocity, Φ is the peak-to-peak flapping amplitude, *f* is the flapping frequency, *R* is the wing length, *S* is the wing surface area, and *C*_L_ is the cycle-averaged lift coefficient in hover (Fig. 1).

Unlike the position, the body pitch angle θ continuously varies during hovering (Fry et al., 2005; Willmott and Ellington, 1997). In the longitudinal plane, the body pitch acceleration is balanced by the aerodynamic pitch moment as:
(2)

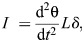
where *I* is the moment of inertia, *t* is the time, and δ is the distance between the center of pressure and the body center of gravity.

A set of corresponding non-dimensional parameters can be found through the Buckingham Π theorem. The lift coefficient *C*_L_ depends on the eight other dimensionless parameters for a hovering animal as:
(3)

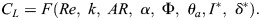
Here, *Re*=ρ*Uc*/µ is the Reynolds number, where µ is the dynamic viscosity of the air and *c*=*S*/*R* is the mean chord length. Subsequently, *k*=π*fc*/*U*=π/(2Φ*AR*) is the reduced frequency, *AR*=*R*^2^/*S*=*R*/*c* is the aspect ratio, α is a representative wing pitch angle, θ_a_ is the body pitch amplitude, I*=*I*/(*mR*_b_^2^) is the non-dimensional moment of inertia of the body where *R*_b_ is the body length, and δ*=δ/*R*_b_ is the non-dimensional moment arm, such that *C*_L_δ* represents a pitching moment coefficient. Because the moment of inertia is evaluated around the pitch rotation axis, we assume the influence of wing inertia to be negligible due to the small wing-to-body mass ratio (Dudley and Ellington, 1990b). Although the wing-to-body mass ratio could reach up to 15% for birds (Xu et al., 2021), it remains significantly lower for normal hovering animals, such as bumblebees (0.18–0.29%), hawkmoths (2–3%), flies (0.12–2.24%), and hummingbirds (3.9%). The wing flexibility effects and associated parameters are not explicitly considered in this study. Note, however, that the observed animal wing angles and hence flight are outcomes of the fluid-structure interaction of the flexible flapping wings.

### Scaling relations

To quantify the relationship between mass *m* and other hover-related quantities *y* for each species, we assume a power law relationship in the form of *y*=β*m*^γ^ with the intercept β and the allometric coefficient (exponent) γ. Each *y* is represented by weighted mean quantities based on reported measurements. This calculation, ‘weighted.mean’, is performed in R (v. 4.2.3) and considers the unique number of observations *N*_ob_ reported for each parameter. These weighted means are then aggregated across the *N*_sp_ species to report the empirical results from OLS regressions γ_OLS_. The OLS regressions are performed via the linear model *lm* function in R, which fits the intercept and slope by minimizing the sum of the squared residuals in the assumed power law relationship.

Phylogenetic covariance is incorporated into the regression analysis to assess the influence of phylogenetic relationship on the scaling exponents. PGLS regression analysis γ_PGLS_ are performed using the *gls* function from the *nlme* package in R. The covariance matrix is calculated using Pagel's λ method (Pagel, 1999; Freckleton et al., 2002) in R (specifically *corPagel* with λ=0.5). The phylogenetic tree (Fig. S1) is constructed by matching the species name to the NCBI taxonomy database and obtaining taxonomy data from an online phylogenetic tree generator (https://phylot.biobyte.de/). Species name changes are documented in Table S1. The resulting γ_OLS_ and γ_PGLS_ are compared to the theoretically derived scaling exponent of γ_theory_.

These data are from literature that report the measured flights of hovering insects and hummingbirds with sufficient information on the morphology (mass *m*, wing length *R*, and wing area *S*) and flapping kinematics (frequency *f* and peak-to-peak amplitude Φ) (Chai and Millard, 1997; Cheng and Sun, 2016; Dudley and Ellington, 1990a; Ellington, 1984a,b; Fry et al., 2005; Groom et al., 2018; Kolomenskiy et al., 2019; Lehmann and Dickinson, 1997; Liu et al., 2024; Meng and Sun, 2015; Willmott and Ellington, 1997) to characterize the aerodynamics of hovering flight.

As far as we know, the considered data set (Data S1) integrates all available experimental data that meet these conditions. The data by Weis-Fogh (1973) were not considered in this study because this dataset comprises mixed pure hover and slow forward flight data without specification. Most data points consolidated in the analysis by Weis-Fogh (1973) are from early 20th century books and journals that we cannot obtain, or undocumented correspondences. This makes it difficult to filter out the data that would be relevant to the present study. In addition, hovering bats show asymmetric hovering (Håkansson et al., 2015; Norberg et al., 1993) and, as such, are excluded from the main study. The application of the proposed scaling model on the extended data (Data S1) including the data by Weis-Fogh (1973) and bats (Håkansson et al., 2015; Norberg et al., 1993) is shown in the supplementary material (Fig. S2 and Tables S2, S3).

## Supplementary Material

10.1242/biolopen.061932_sup1 Supplementary information

Dataset 1. Excel table listing the considered experimental observations of the morphology and kinematics of normal hovering animals from the literature. The extended dataset includes the main data in addition to the data by Weis-Fogh (1973) and bats (Håkansson et al., 2015; Norberg et al., 1993). The data by Weis-Fogh (1973), Håkansson et al. (2015) and Norberg et al. (1993) includes mixed-mode flight data and as such excluded in the main study. Also note that the atmospheric density values are calculated from the reported measurement altitudes using a 1974 standard atmosphere calculator. These densities are then used to capture altitude-based effects in the calculation of lift coefficient and Reynolds number. If no altitudes nor atmospheric densities were reported in the original study, the standard sea-level density was assumed.
