# Supplementary material for: Deciphering the flapping frequency allometry: unveiling the role of sustained body attitude in the aerodynamic scaling of normal hovering animals
Source: Biol Open. 2025 Mar 14;14(3):bio061932. doi: 10.1242/bio.061932 (PMC11928051; doi:10.1242/bio.061932)
Supplement: Supplementary information [file biolopen-14-061932-s1.pdf]

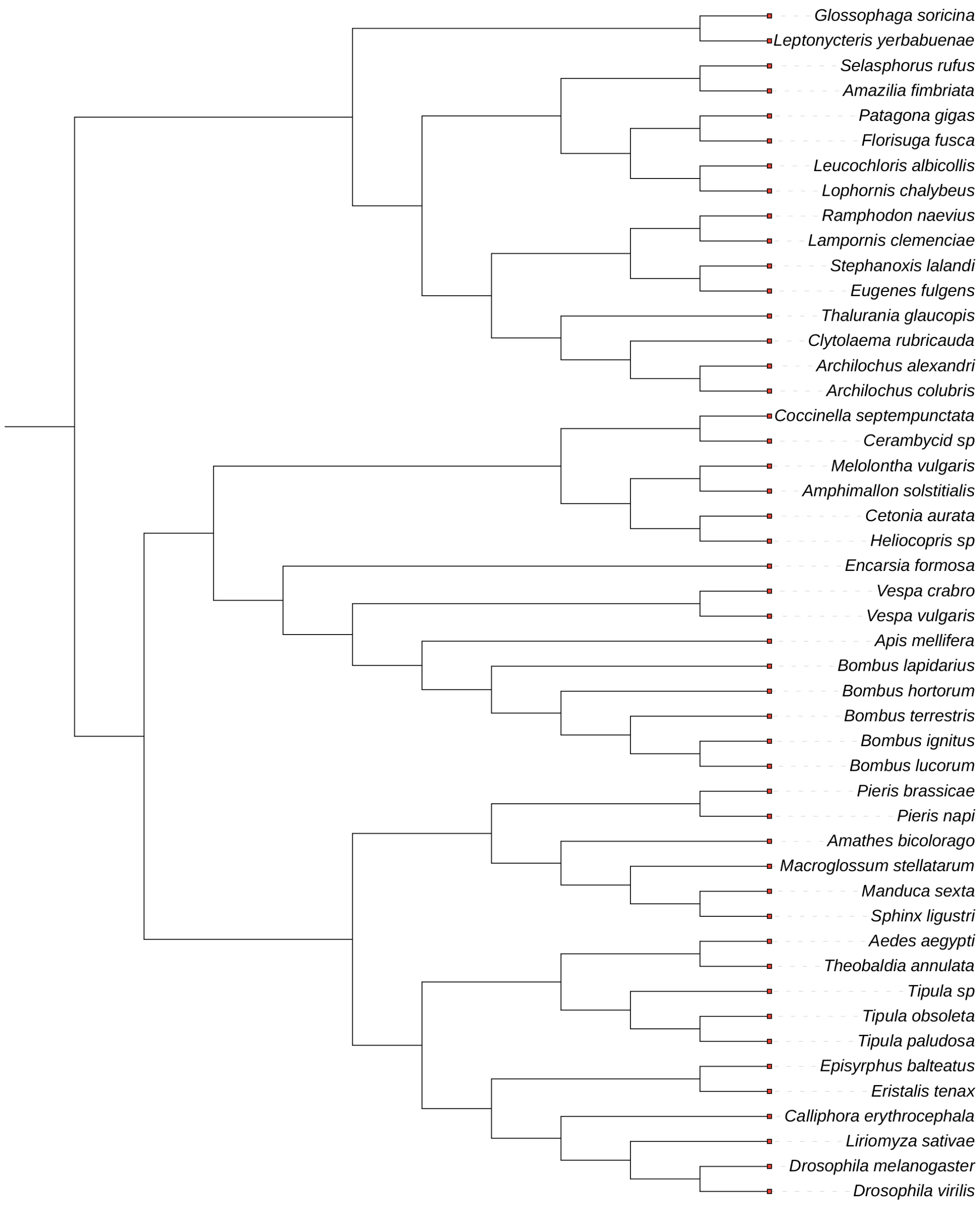

Fig. S1. Phylogenetic tree. It is created using the Interactive Tree of Lift tool at https://itol.embl.de/.

Biology Open • Supplementary information

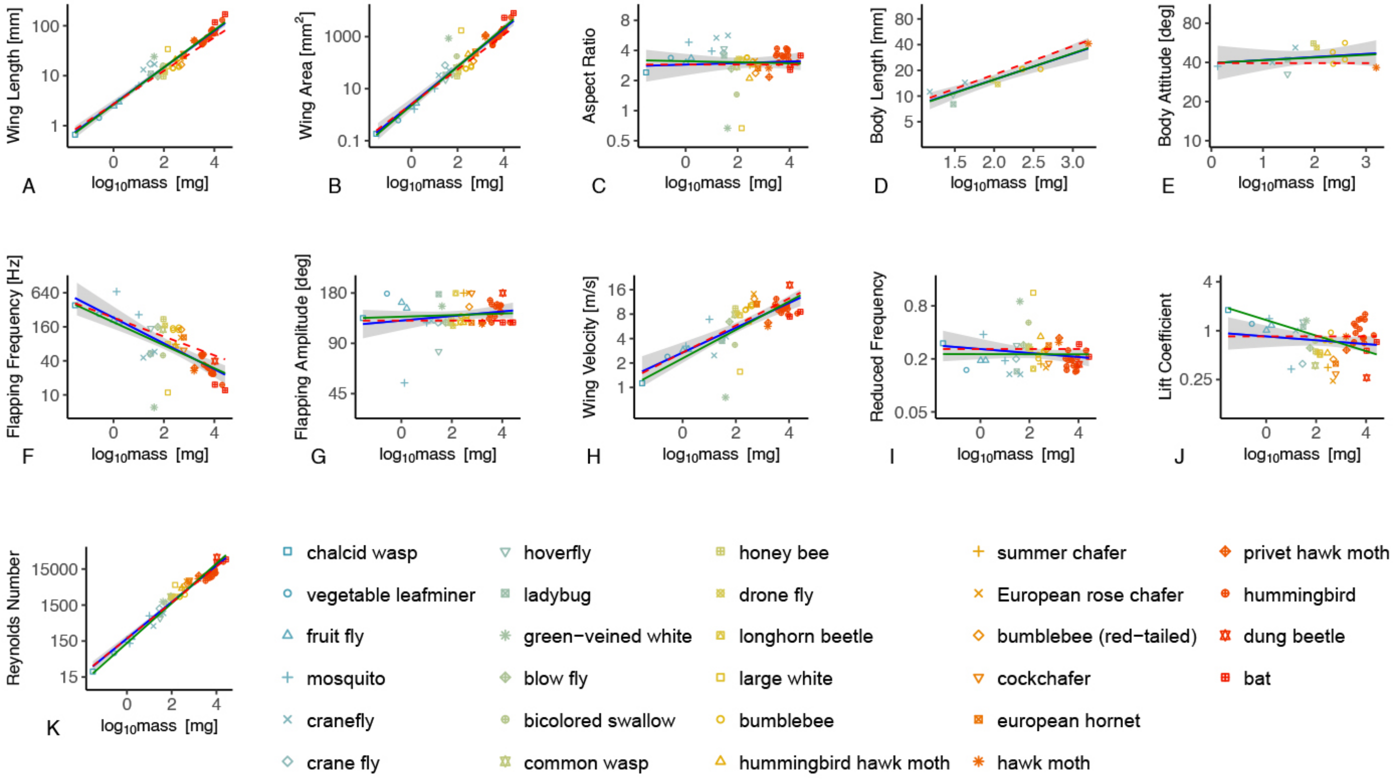

Fig. S2. Considered animal flight observations from the extended data and the ordinary least-squares (OLS) and phylogenetic generalized least-squares (PGLS) regressions for a) the wing length R, b) wing area S, c) aspect ratio AR, d) body length R_b, e) body attitude (mean body pitch angle θ_m), f) flapping frequency f, g) flapping amplitude Φ, h) wing velocity U, i) reduced frequency k, j) lift coefficient C_L, and k) Reynolds number Re with mass m. Shaded regions: 95% confidence intervals for OLS regression. Blue and green solid lines: OLS and PGLS regression. Red dashed lines: theoretical scaling. Table S2 and Table S3 show the numeric results.

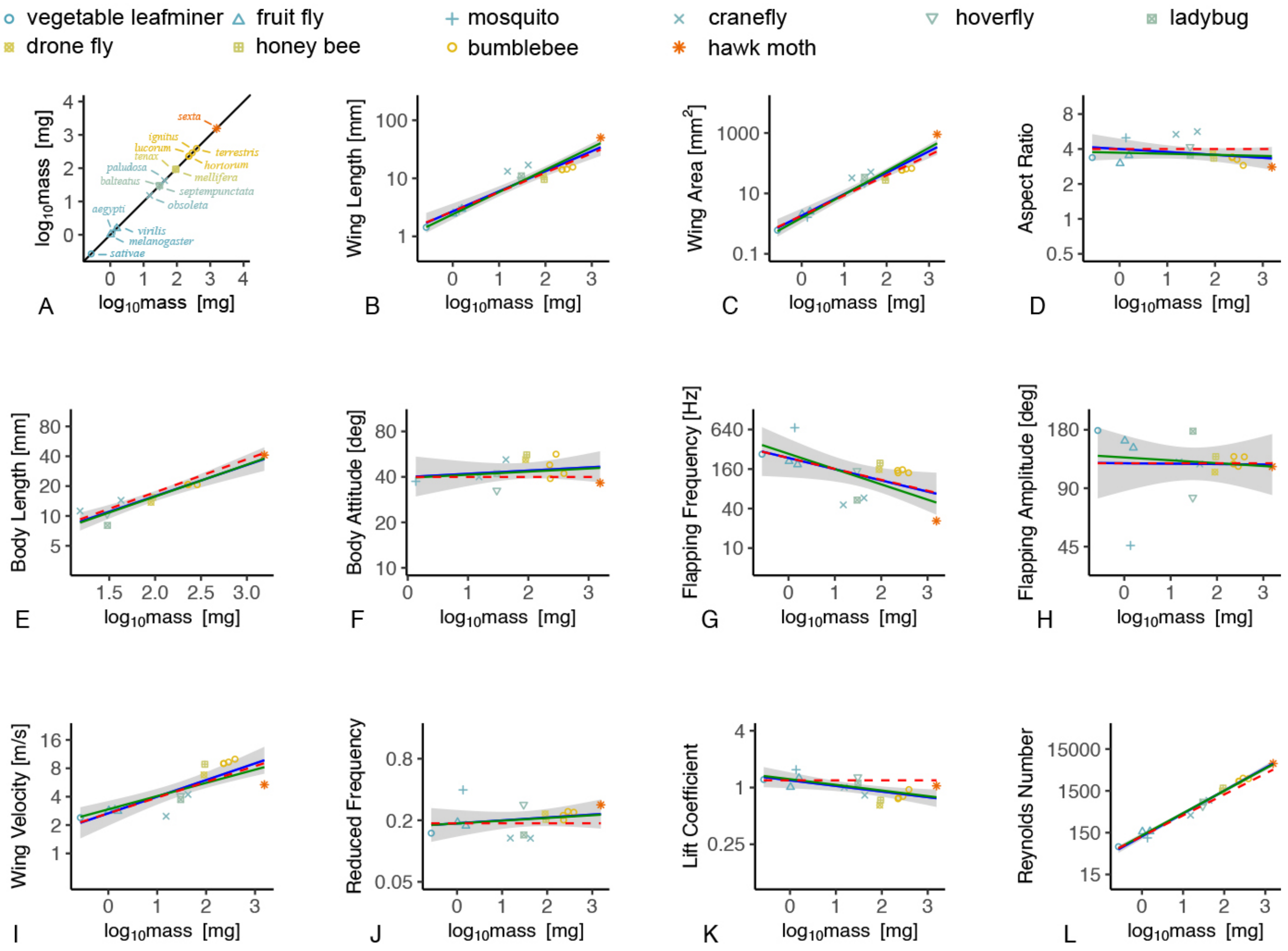

Fig. S3. The OLS and PGLS regressions for b) the wing length R, c) wing area S, d) aspect ratio AR, e) body length R_b, f) mean body pitch angle θ_m, g) flapping frequency f, h) flapping amplitude Φ, i) wing velocity U, j) reduced frequency k, k) lift coefficient C_L, and l) Reynolds number Re with mass m for the normal hovering animals in the main data without the hummingbirds. Shaded regions: 95% confidence intervals for OLS regression. Blue and green solid lines: OLS and PGLS regression. Red dashed lines: theoretical scaling. Table S4 and Table S5 show the numeric results.

Table S1. List of species name replacements. *The NCBI taxonomy did not include *Tipula_obsoleta*, which was replaced by Tipula oleracea to generate the phylogenetic tree.

Current study	NCBI
Amathes_bicolorago	*Agrochola_bicolorago*
Calliphora_erythrocephala	*Calliphora_vicina*
Theobaldia_annulata	*Culiseta_annulata*
Tipula_sp	*Tipula_sp*
*Tipula_obsoleta**	*Tipula_oleracea*
Vespa_vulgaris	*Vespula_vulgaris*
Amphimallon_solstitialis	*Amphimallon_solstitiale*
Melolontha_vulgaris	*Melolontha_melolontha*
Heliocopris_sp	*Heliocopris_sp._sp67*
Cerambycid_sp	*Cerambycidae_sp.*
Amazilia_fimbriata	*Chionomesa_fimbriata*

Table S2. Theoretical and empirical OLS scaling results for the extended dataset including mixed-mode flight data from Weis-Fogh (1973), and bats (Håkansson et al., 2015; Norberg et al., 1993). R: wing length, S: wing area, AR: aspect ratio, R_b: body length, θ_m: body attitude (mean body pitch angle), f: flapping frequency, Φ: flapping amplitude, U: wing velocity, k: reduced frequency, C_L: lift coefficient, and Re: Reynolds number.

	N_{ob}	N_{sp}	γ_{theory}	γ_{OLS}	$\log_{10}\beta$	r^2	p-value	95% CI
R	204	48	1/3	0.366	0.431	0.936	< 0.001	[0.338, 0.395]
S	204	48	2/3	0.724	0.406	0.883	< 0.001	[0.647, 0.801]
AR	204	48	0	0.008	0.461	-0.018	0.686	[-0.030, 0.045]
R_b	42	10	1/3	0.304	0.585	0.855	< 0.001	[0.209, 0.400]
θ_m	30	12	0	0.024	1.597	-0.036	0.452	[-0.043, 0.090]
f	176	48	-1/6	-0.228	2.366	0.477	< 0.001	[-0.297, -0.159]
Φ	176	48	0	0.014	2.090	0.018	0.179	[-0.006, 0.034]
U	176	48	1/6	0.153	0.430	0.502	< 0.001	[0.109, 0.197]
k	176	48	0	-0.023	-0.587	0.005	0.271	[-0.063, 0.018]
C_L	176	48	0	-0.024	-0.070	0.0004	0.318	[-0.071, 0.023]
Re	176	48	1/2	0.507	2.245	0.954	< 0.001	[0.474, 0.540]

Biology Open • Supplementary information

Table S3. Theoretical and empirical PGLS scaling results for the extended dataset including mixed-mode flight data from Weis-Fogh (1973), and bats (Håkansson et al., 2015; Norberg et al., 1993). *R*: wing length, *S*: wing area, *AR*: aspect ratio, R_b: body length, θ_m: body attitude (mean body pitch angle), *f*: flapping frequency, Φ: flapping amplitude, *U*: wing velocity, *k*: reduced frequency, C_L: lift coefficient, and *Re*: Reynolds number.

	N_{ob}	N_{sp}	γ_{theory}	γ_{PGLS}	$\log_{10}\beta$	*p*-value
R	204	48	1/3	0.371	0.429	< 0.001
S	204	48	2/3	0.740	0.388	< 0.001
AR	204	48	0	0.0005	0.476	0.983
R_b	42	10	1/3	0.308	0.573	< 0.001
θ_m	30	12	0	0.025	1.587	0.508
f	176	48	-1/6	-0.188	2.234	< 0.001
Φ	176	48	0	0.002	2.122	0.912
U	176	48	1/6	0.187	0.323	< 0.001
k	176	48	0	-0.003	-0.636	0.913
C_L	176	48	0	-0.110	0.170	< 0.001
Re	176	48	1/2	0.558	2.107	< 0.001

Table S4. Theoretical and empirical OLS scaling results for the dataset without hummingbird. *R*: wing length, *S*: wing area, *AR*: aspect ratio, R_b: body length, θ_m: body attitude (mean body pitch angle), *f*: flapping frequency, Φ: flapping amplitude, *U*: wing velocity, *k*: reduced frequency, C_L: lift coefficient, and *Re*: Reynolds number.

	N_{ob}	N_{sp}	γ_{theory}	γ_{OLS}	$\log_{10}\beta$	r^2	*p*-value	95% CI
R	102	15	1/3	0.346	0.431	0.880	< 0.001	[0.273, 0.419]
S	102	15	2/3	0.710	0.265	0.914	< 0.001	[0.584, 0.835]
AR	102	15	0	-0.025	0.602	0.017	0.284	[-0.074, 0.024]
R_b	42	10	1/3	0.314	0.572	0.876	< 0.001	[0.224, 0.404]
θ_m	30	12	0	0.021	1.602	-0.050	0.508	[-0.047, 0.090]
f	75	15	-1/6	-0.171	2.373	0.237	0.038	[-0.330, -0.011]
Φ	75	15	0	-0.002	2.083	-0.077	0.955	[-0.081, 0.076]
U	75	15	1/6	0.176	0.426	0.665	< 0.001	[0.105, 0.248]
k	75	15	0	0.028	-0.729	-0.018	0.402	[-0.042, 0.099]
C_L	75	15	0	-0.059	0.076	0.329	0.015	[-0.105, -0.014]
Re	75	15	1/2	0.545	2.088	0.986	< 0.001	[0.507, 0.583]

Table S5. Theoretical and empirical PGLS scaling results for the dataset without hummingbird. *R*: wing length, *S*: wing area, *AR*: aspect ratio, R_b: body length, θ_m: body attitude (mean body pitch angle), *f*: flapping frequency, Φ: flapping amplitude, *U*: wing velocity, *k*: reduced frequency, C_L: lift coefficient, and *Re*: Reynolds number.

	N_{ob}	N_{sp}	γ_{theory}	γ_{PGLS}	$\log_{10}\beta$	*p*-value
R	102	15	1/3	0.384	0.379	< 0.001
S	102	15	2/3	0.772	0.193	< 0.001
AR	102	15	0	-0.009	0.570	0.709
R_b	42	10	1/3	0.321	0.553	< 0.001
θ_m	30	12	0	0.021	1.596	0.589
f	75	15	-1/6	-0.233	2.436	0.016
Φ	75	15	0	-0.015	2.113	0.743
U	75	15	1/6	0.140	0.468	< 0.001
k	75	15	0	0.027	-0.730	0.539
C_L	75	15	0	-0.060	0.088	0.047
Re	75	15	1/2	0.534	2.109	< 0.001

Biology Open • Supplementary information

Dataset 1. Excel table listing the considered experimental observations of the morphology and kinematics of normal hovering animals from the literature. The extended dataset includes the main data in addition to the data by Weis-Fogh (1973) and bats (Håkansson et al., 2015; Norberg et al., 1993). The data by Weis-Fogh (1973), Håkansson et al. (2015) and Norberg et al. (1993) includes mixed-mode flight data and as such excluded in the main study. Also note that the atmospheric density values are calculated from the reported measurement altitudes using a 1974 standard atmosphere calculator. These densities are then used to capture altitude-based effects in the calculation of lift coefficient and Reynolds number. If no altitudes nor atmospheric densities were reported in the original study, the standard sea-level density was assumed.

Available for download at
https://journals.biologists.com/bio/article-lookup/doi/10.1242/bio.061932#supplementary-data
